# Flow-Based CL-SMIA for the Quantification of Protein Biomarkers from Nasal Secretions in Comparison with Sandwich ELISA

**DOI:** 10.3390/bios13070670

**Published:** 2023-06-22

**Authors:** Julia Neumair, Marie Kröger, Evamaria Stütz, Claudia Jerin, Adam M. Chaker, Carsten B. Schmidt-Weber, Michael Seidel

**Affiliations:** 1 Chair of Analytical Chemistry and Water Chemistry, TUM School of Natural Sciences, Technical University of Munich, Lichtenbergstr. 4, 85748 Garching, Germany; julia.neumair@tum.de (J.N.); marie.kroeger@tum.de (M.K.); 2 Center of Allergy and Environment (ZAUM), Technical University of Munich and Helmholtz Center Munich, Member of the German Center of Lung Research (DZL), 80802 Munich, Germany; evamaria.stuetz@tum.de (E.S.); claudia.jerin@tum.de (C.J.); adam.chaker@tum.de (A.M.C.); csweber@tum.de (C.B.S.-W.); 3 TUM School of Medicine, Department of Otorhinolaryngology, Klinikum Rechts der Isar, Technical University of Munich, 81675 Munich, Germany

**Keywords:** biomarker, microarray, interferon, ELISA, microfluidic, chemiluminescence, nasal secretion

## Abstract

Protein biomarkers in nasal secretions can be used as a measure to differentiate between allergies, airway diseases and infections for non-invasive diagnostics. The point-of-care quantification of biomarker levels using flow-based microarray facilitates precise and rapid diagnosis and displays the potential for targeted and effective treatment. For the first time, we developed a flow-based chemiluminescence sandwich microarray immunoassay (CL-SMIA) for the quantification of nasal interferon-beta (IFN-β) on the Microarray Chip Reader-Research (MCR-R). Polycarbonate foils are used as a cost-effective surface for immobilizing capture antibodies. By using a commercially available set of anti-human IFN-β antibodies, the CL-SMIA can be compared directly to an enzyme-linked immunosorbent assay (ELISA) performed in microtiter plates concerning the bioanalytical performance and economic issues. Pre-incubation of the sample with detection antibodies facilitates the lower consumption of detection antibodies, as this allows for a longer interaction time between the antibody and the biomarker. The direct injection of pre-incubated samples into the microarray chips eliminates the adsorption of proteins in the tubing as well as the contamination of the tubing and valves of the MCR-R with clinical samples. The small flow cell allows for a low sample volume of 50 μL. The limit of detection of 4.53 pg mL^−1^ was slightly increased compared to a sandwich ELISA performed on microtiter plates which were 1.60 pg mL^−1^. The possibility to perform the CL-SMIA in a multiplexed mode makes it a promising assay for the rapid and cost-effective non-invasive detection of biomarkers in nasal secretions.

## 1. Introduction

Biomarkers are determinable biological indicators for complex processes in the human body and can be used to monitor different diseases of the upper and lower airways. Furthermore, they can facilitate the measurements of physiologic parameters such as blood sugar but also proteins or enzymes [1,2]. Body fluids are a great source of protein biomarkers [3], whereby non-invasive sampling is preferred, for example for urine and nasal secretions. The in nasal secretions pattern of biomarkers may indicate whether a patient’s symptoms in the lower airways are caused by allergies or infections, and treatment can be adjusted accordingly [4,5]. Interferons, a group of class II cytokines, herby play a great role in the immune response during viral infections. Specifically, IFN-β production is enhanced upon infection [6], making it a suitable biomarker for viral infections. 

The detection and quantification of cytokines mostly rely on immuno-based methods [7,8], but also methods such as aptamer-based assays [9] or mass spectrometry [10] are used. Among the immunoassays, methods such as ELISAs (enzyme-linked immunosorbent assay) [7,11] and flow cytometry [12,13], as well as biosensors, are common. The latter utilizes different detection methods such as label-free approaches, including SPR (surface plasmon resonance) [14], or labeling with fluorescent [15] or chemiluminescent [16] labels. 

For microarray assays, multiple catchers are immobilized on the microarray surface, enabling simultaneous detection of multiple analytes in one single measurement [17,18]. Microarrays can be performed in a good format [8,19,20], but the use of a chip or chip-like design holds the opportunity of simplifying the assay by allowing for flow-based approaches [21,22].

In this work, we developed a flow-based, chemiluminescence sandwich microarray immunoassay (CL-SMIA) for the quantification of IFN-β from nasal secretions on the Microarray Chip Reader-Research (MCR-R). The MCR-R is a flow-based biosensor platform for CL detection via antibody microarray chips, where the reagents are directed automatically over the flow cell of microarray chips. The volumes and flow rates of these reagents can be adjusted as needed, which makes them much more flexible for assay optimization compared to static incubated immunoassay formats [23]. Features of this newest version of MCRs are described in detail elsewhere, where it has also been shown that the MCR-R can be used for diagnostic testing [24]. Using polycarbonate (PC) as a surface enables more cost-effective and faster production of microarray chips compared to glass surfaces [24,25]. Additionally, PC is available as flexible foil, which requires not only fewer resources but also handling can be simplified. Processes such as roll-to-roll fabrication can furthermore enhance the fabrication of microarray platforms [26]. 

This proof-of-concept study showed the feasibility of using foil-based microarray chips together with antibodies from a commercially available ELISA antibody kit for the detection of IFN-β. Before automated CL detection on the MCR-R, the sample is pre-incubated with the detection antibody and is then injected into the microarray chip. This allows for a low sample volume of 50 μL, as well as the possibility to stagger the measurements in time and, therefore, increase the sample quantity. Calibration of the spiked IFN-β in diluted nasal secretions revealed a slightly increased, but similar detection limit, compared to a sandwich ELISA performed with the same antibody kit. Therefore, we see the CL-SMIA, with its ability for multiplexing, as a promising method for rapid and cost-effective point-of-care determination of biomarker levels. 

## 2. Materials and Methods

### 2.1. Material and Buffers

If not stated otherwise, the chemicals were purchased from Sigma Aldrich (Darmstadt, Germany), a subsidiary of Merck, or Carl Roth (Karlsruhe, Germany). The R&D Systems Human IFN-β DuoSet ELISA containing capture (CAB) and detection antibodies (DAB) for IFN-β, recombinant human IFN-β standards, as well as horseradish-peroxidase-labeled streptavidin (strep-HRP) and the substrate reagent kit for ELISA detection, were purchased from Bio-Techne (Wiesbaden, Germany). The assay buffer (Ab-33k), used for sample collection and as a sample matrix, was purchased from Merck Millipore (Darmstadt, Germany). The CL-reagents (luminol and hydrogen peroxide) were used from the Elistar Supernova reagent kit from Cyanagen (Bologna, Italy). The Strep-HRP, for the CL-SMIA, was purchased from Biozol (Eching, Germany) and the polycarbonate foils (Makrolon^®^ GP, 0.25 mm) from Modulor (Berlin, Germany). Ultrapure water was used unless stated otherwise.

Phosphate buffered saline (PBS, pH 7.4) consisted of 137 mM NaCl, 2.7 mM KCl, 8.1 mM Na_2_HPO_4_ and 1.5 mM KH_2_PO_4_. The washing buffer, used for the ELISA experiments, was a 0.05% Tween^®^ 20 solution in PBS and the running buffer, for the CL-SMIA, was a 0.1% Tween^®^ 20 solution in PBS. The spotting buffer, used for microcontact printing, was prepared using 100 mg mL^−1^ trehalose dihydrate, 2 mg mL^−1^ 1-ethyl-3-(3-(dimethylamino)propyl)carbodiimide (EDC), 2 mg mL^−1^ N-hydroxysulfosuccinimide sodium salt (sNHS) and 0.02 mg mL^−1^ Pluronic^®^ F-127.

### 2.2. Collection of Real Nasal Mucosal Lining Fluid Samples

Nasal mucosal lining fluid samples were collected during an ongoing clinical validation study. The “Airway Disease Analysis and Prevention” (ADAPT) study is an EIT Health-funded prospective observational biomarker study (funding number 19065) involving four academic and two industrial study partners. The nasal mucosal lining fluid samples used for this study have been exclusively collected at the Klinikum rechts der Isar of the Technical University of Munich (IRB approval 159/19S).

Nasal mucosal lining fluid samples were collected using Nasosorption^TM^ FX-I sampling devices (Hunt Developments Ltd., Midhurst, UK), as previously described [27]. Briefly, sampling was performed by carefully inserting one Nasosorption device into both nostrils for 60 s, while gently pressing on the ala nasi (wing of the nose), to collect the nasal mucosal lining fluid from the mucosal surface of the lumen of each nostril. The nasal samples were then eluted in a 300 μL assay buffer (Ab-33k) and centrifuged at 16,000 × *g* at 4 °C for 20 min. The samples were stored at −70 °C until further use.

The initial IFN-β concentrations of different biomarkers were measured with the MSD Mesoscale platform Meso QuickPlex SQ120 MM (Meso Scale Discovery, Inc., Rockville, MD, USA) using U-PLEX Custom Biomarker (hu) Assays and SECTOR for IFN-β, according to the manufacturer’s protocol. Samples with the lowest IFN-β levels were chosen and the IFN-β standards were added to reach the desired IFN-β concentrations.

### 2.3. Sandwich ELISA

#### Assay

For the ELISA experiments, the protocol given by the manufacturer for the DuoSet was followed with minor changes. Experiments were conducted in MICROLON^®^ 600, high binding, polystyrene 96-well plates with a flat bottom from Greiner Bio-One (Frickenhausen, Germany). In the following, the volumes are always meant for one well. First, a 100 μL of a 2.0 μg mL^−1^ solution of anti-human IFN-β CAB in PBS were incubated over night at room temperature (RT). Afterwards, the ELISA plate was washed three times with a 400 μL washing buffer, each with the ELx405 Select plate washer from BioTek, Winooski, VT, USA. Residual active sites were blocked with 300 μL of a 1% casein solution in PBS for 1 h. After washing, 100 μL of the sample was added, as well as 15 μL of a 7.7% bovine serum albumin (BSA) solution in PBS to reach a 1% BSA concentration, as suggested by the manufacturer’s protocol. Incubation took place for 2 h at RT at 100 rpm. A total of 100 μL of a 62.5 ng mL^−1^ anti-human IFN-β DAB solution, in 1% BSA in PBS, were added after another washing and incubated for 2 h at 100 rpm. Again, the plate was washed and 100 μL of a 40-fold dilution of the strep-HRP, contained in the DuoSet, was incubated at RT for 20 min at 100 rpm. After the last washing step, 100 μL of substrate solution (mixed from the substrate reagent kit according to its instructions) was incubated at RT in the dark until a baby blue colour developed or for a maximum of 20 min. In total, 50 μL of a 1 M sulfuric acid solution was added to stop the colour reaction and the plate was measured at 450 and 540 nm using a Synergy HT plate reader. For wavelength correction, the absorbance values at 540 nm were subtracted from the ones at 450 nm.

### 2.4. Flow-Based CL-SMIA 

#### 2.4.1. Preparation of Microarray Chips 

**Figure 1:**
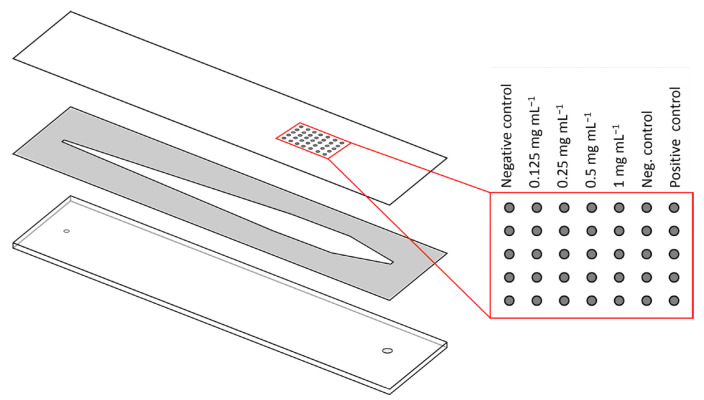
Schematic representation of the microarray chip setup. The polycarbonate chip with immobilized antibodies (**top**) is adhered with a double-sided adhesive with a cut-out flow channel (**middle**) to the polyoxymethylene carrier (**bottom**). On the right, the spotting scheme for optimization and calibration experiments is shown.

As the surface for the microarray chips, PC foils were used and prepared similarly as described elsewhere [25]. In short, a CE 6000–40 cutting plotter from Graphtec Corporation (Yokohama, Japan) was used to cut the PC foil (0.25 mm) into sheets of 3 × 3 chips, each having a size of 26 × 76 mm with one pass. The device was adjusted to a cut force of 18 au., with a speed of 15 cm s^−1^ and acceleration of 1 au. For functionalization, a succinylated Jeffamine^®^ ED-2003 was applied using a screen printer and incubated for 2 h at 95 °C. After washing and drying, the sheets were stored at RT under reduced humidity until further use. Anti-human IFN-β CAB, from the ELISA DuoSet, was immobilized via contact spotting using a BioOdyssey Calligrapher^®^ MiniArrayer from Bio-Rad (Hercules, CA, USA). Rows of five spots, with distances of 1300 μm between the rows and 1100 μm between the spots of one row (spot size 150 μm), were generated. CAB solutions of different concentrations were mixed 1:1 with the spotting buffer, leading to end concentrations of 0.125, 0.25, 0.5 and 1 mg mL^−1^. As a positive control, polyclonal anti-peroxidase antibodies from a rabbit (1:40 dilution final) and PBS as negative control were mixed 1:1 with the spotting buffer. In Figure 1, the spotting scheme for optimization and calibration experiments using all four CAB concentrations is shown. For recovery experiments, only 0.125 mg mL^−1^ was immobilized. A temperature of 20 °C and 55% relative humidity were used for spotting and incubating for 1 h. Afterwards, the sheets were separated into individual chips, which were stored at 4 °C until further use. A double-sided adhesive with a cut-out flow channel (thickness 140 μm, channel size 56 μL) was used to combine the PC chips with black polyoxymethylene carriers with in-and outlets to the finished microarray chips (Figure 1).

#### 2.4.2. Measurements with Flow-Based CL-SMIA

The CL-SMIA was conducted on the MCR-R built by GWK Präzisionstechnik (Munich, Germany). To prepare the device at the beginning of a measuring day, all tubes were filled with the running buffer. Additionally, all reagents used for the assay, namely a dilution of strep-HRP in the running buffer and the individual CL reagents, were loaded and the microarray chip holder was heated to 37 °C. 

For every microarray chip, first, a dark frame picture was taken by inserting it into the microarray chip holder, flushing it with a running buffer and recording an image for 60 s without any addition of CL reagents. Parameters that were optimized in this work are shown in Table 1. For measurements, 50 μL of the sample in the assay buffer was mixed with 8.75 μL of 6.9% BSA (final concentration 1%) and 1.25 μL biotinylated anti-human IFN-β DAB from the ELISA DuoSet in a reaction tube and pre-incubated at 37 °C and 100 rpm. Following this, the mixture was injected manually into the microarray chip using a pipette and incubated again. After reinserting the microarray chip into the MCR-R, the chip was flushed, first slowly (50 μL, 0.5 μL s^−1^), then faster (1500 μL, 325 μL s^−1^) with a running buffer. The Strep-HRP solution was passed over the chip (2.0 μL s^−1^) and after another washing step (1500 μL, 325 μL s^−1^), the CL reagents were delivered over the chip in a 1:1 ratio (200 μL each, 100 μL s^−1^). Immediately, the CCD camera recorded an image for 60 s. After every measurement, the tubing was flushed with the running buffer. Table 2 shows the detailed measuring program on the MCR-R. 

### 2.5. Data Evaluation

For every measurement, the software on the MCR-R automatically subtracted the CL signals of the dark frame from the CL signals of the actual measurement. The software MCR spotreader (Stefan Weißenberger, Munich, Germany) was used to evaluate the resulting CL signals by placing a grid over the image, resulting in one spot per box. Automatically, for every spot, a mean of the 10 brightest pixels was calculated. For every row of five spots corresponding to the antibody concentrations or controls, the mean was calculated omitting spots that deviated more than 10%. 

## 3. Results and Discussion 

### 3.1. ELISA 

#### Calibration Curve

**Figure 2:**
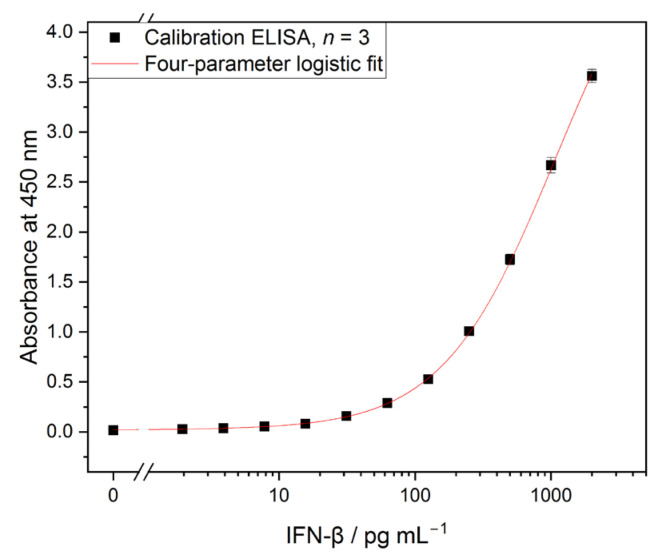
Calibration curve of ELISA for IFN-β *(n* = 3). Limit of detection (LOD) 1.60 pg mL^−1^, median effective concentration (EC50) 1082 pg mL^−1^.

Before calibration of the anti-human IFN-β ELISA was performed, the assay was optimized regarding blocking agent and antibody concentrations (Supplementary Materials Table S1). For the calibration curve, the wavelength corrected absorbance at 450 nm was plotted semi-logarithmically (*y*-axis linear, *x*-axis logarithmic) versus the concentration of human IFN-β standard and fitted with a four-parameter logistic fit (Equation (1)). The calibration curve is shown in Figure 2. A limit detection (LOD, Equation (2)) of 1.60 pg mL^−1^ and a median effective concentration (EC50) using Equation (1) of 1082 pg mL^−1^ were obtained.

(1) y=Amin+Amax−Amin1+x0xh

*y* represents the measured adsorption at IFN-β standard concentration, *x*. *A_min_* refers to the minimum asymptote, or the response of the sample, with no standard. *A_max_* is the maximum asymptote, or the response of the samples, with a very high standard concentration approach. *x*_0_ is assigned the inflection point at which the curve changes direction and *h* is the slope of the curve. For curves without *A_max_*, a reasonable upper limit was estimated by the software, still providing valuable measures.

(2) $$\mathrm{LOD}\;=\;\mathrm{mean}\;\mathrm{absorbance}\;\left(\mathrm{blank}\right)+3\times \;\mathrm{standard}\;\mathrm{deviation}\;\left(\mathrm{blank}\right)$$

### 3.2. Flow-Based CL-SMIA

Microarray chips for the CL-SMIA were produced using PC foils with a thickness of 0.25 mm [28,29]. In prior studies, PC plates with a thickness of 1 mm were used [24,25,30]. Thinner material has the advantage of easier and faster processing with the cutting plotter, as fewer passes of the blade make the process faster. Additionally, the separation of the individual chips, from the 3 × 3 plate, is easier as breaking the thicker plates apart requires some strength and the foils can be cut using scissors. PC foils also hold the opportunity for roll-to-roll fabrication in the future.

CABs are immobilized via their free primary amines to the free carboxy groups on the chip surface by using EDC and sNHS. Adding the coupling reagents into the spotting solution only activates the surface where needed, which reduces unspecific binding and, therefore, makes blocking unnecessary. The small spot diameter of 150 μm and the corresponding volume of 0.9 nL require only small amounts of CAB per row of spots on the microarray chip (0.6 ng), which reduces the costs for antibody microarrays compared to ELISA (200 ng).

For the flow-based CL-SMIA, the sample and DAB were first pre-incubated in a reaction tube to form an equilibrium of the IFN-β-DAB complex before injecting the mixture directly into the flow cell of the microarray chip using a pipette. In this way, the DAB and sample do not have to pass the tubes and valves of the MCR-R, which reduces the adsorption of both and allows for smaller volumes. Contamination of the tubes with the samples, and any possible carryover, was avoided as well. Additionally, this allowed the experiments to be staggered in time, which increases throughput.

**Figure 3:**
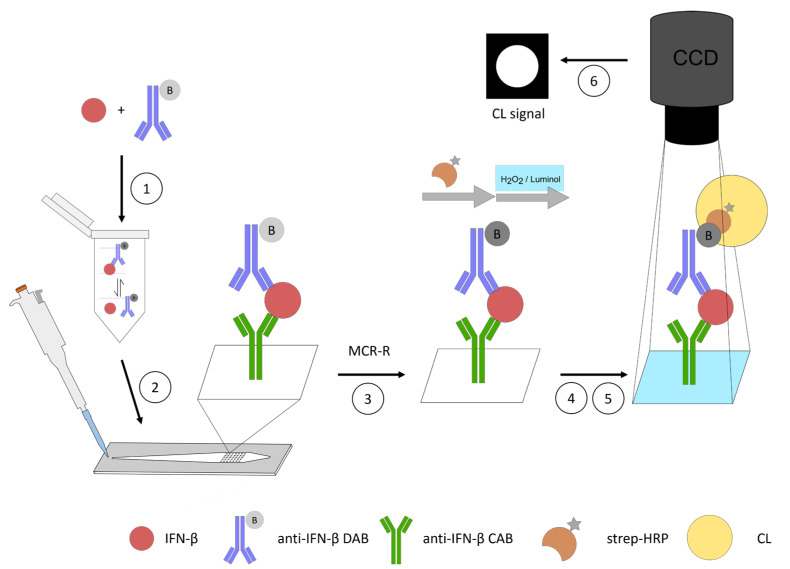
Schematic representation of the flow-based CL-SMIA. (1): pre-incubation of the sample and anti-human interferon beta (IFN-β) detection antibody (DAB). (2): Sample injection into the flow cell of the microarray chip and on-chip incubation and interaction of the IFN-β-DAB complex with immobilized anti-human IFN-β capture antibody (CAB). (3): Sample delivery in a flow cell of the microarray chip on MCR-R (Microarray Chip Reader-Research). (4): Streptavidin-horseradish peroxidase (strep-HRP). (5): CL reagents delivery over the chip. (6): Acquisition of image.

After on-chip incubation, the microarray chip was inserted into the MCR-R, where the running buffer was delivered slowly (0.5 μL s^−1^) over the chip to slowly wash away the injected sample and to allow for further interactions. After further washing, detection took place in an automated way by flushing strep-HRP over the microarray chip, where it bound to the biotin tag of the DABs. Next, CL reagents luminol and hydrogen peroxide were flushed over the chip. The CL reaction was catalyzed in the places where the strep-HRP had bound. The assay principle is depicted in Figure 3.

#### 3.2.1. Optimization

First, the protocol for IFN-β detection using the CL-SMIA was optimized with an IFN-β concentration of 125 pg mL^−1^. The dilution of strep-HRP, incubation time in the tube and flow cell of the microarray chip and the flow rate for sample delivery over the flow cell, as well as the concentration of DAB, were investigated. In Table 1, the parameters for the respective optimization steps are shown. Four different concentrations of immobilized CAB (0.125, 0.25, 0.5 and 1 mg mL^−1^) were used.

The concentration of strep-HRP was optimized using three different dilutions in the running buffer (1:1500, 1:2000 and 1:2500, Figure 4). The highest CL signals were detected for the lowest dilution, thus, the highest concentration of strep-HRP (5672 ± 985 for 0.125 mg mL^−1^ CAB). This was expected, as a higher amount of strep-HRP enables more bound protein and, therefore, a higher signal. Moreover, the signals for negative control increased with a higher strep-HRP concentration from 259 ± 44 for 1:2500 to 493 ± 180 (both for 0.125 mg mL^−1^ CAB). Therefore, the signal-to-control ratio (SCR) was calculated using Equation (3).

(3) $$\mathrm{SCR}\;\left(\mathrm{CAB}\;\mathrm{concentration}\right)=\frac{\mathrm{mean}\;\mathrm{CL}\;\mathrm{signal}\;\left(\mathrm{spotted}\;\mathrm{CAB}\;\mathrm{concentration}\right)}{\mathrm{mean}\;\mathrm{CL}\;\mathrm{signal}\;\left(\mathrm{spotted}\;\mathrm{negative}\;\mathrm{control}\right)}$$

Hereby was found that the 1:2500 dilution yielded the best signal-to-control ratios (14.6 ± 1.6 for 0.125 mg mL^−1^ CAB). Moreover, this strep-HRP dilution showed the lowest standard deviations for SCR with 8.9 ± 4.7% compared to 16.8 ± 1.8% and 34.9 ± 5.3% for the 1:2000 and 1:1500 ratios, respectively. In conclusion, a dilution of 1:2500 was chosen for further experiments. Over the CAB concentration range, the lower concentration led to lower signals and signal-to-control ratios. This indicates that an excessive amount of CAB on the microarray chip leads to a steric hindrance for the IFN-β-DAB complex.

**Figure 5:**
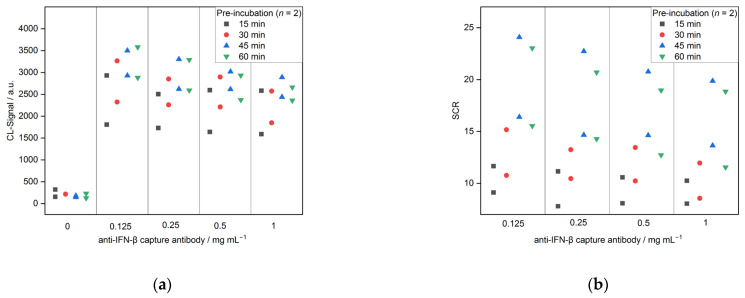
Optimization of pre-incubation step for CL-SMIA (*n* = 2): (**a**) CL-signals and (**b**) SCRs.

Secondly, the pre-incubation of the sample and DAB before injecting it into the flow cell of the microarray chip was optimized in terms of incubation time. Four different times (15, 30, 45 and 60 min) were tested, the results are depicted in Figure 5. Between 15 and 45 min, an increased incubation time led to an increase in the CL signal from 1808 and 2930 for 15 min to 2930 and 3500 for 45 min (both for 0.125 mg mL^−1^ CAB), as well as SCRs from 11.7 and 9.1 to 16.4 and 24.1, respectively. Therefore, with longer incubation times, more IFN-β-DAB complexes seem to be formed. For 60 min, the signals stayed in the same range, as for 45 min (2882 and 3580 for 0.125 mg mL^−1^ CAB), indicating that the equilibrium of complex formation was reached. Therefore, 45 min was chosen as the incubation time for the pre-incubation step.

**Figure 6:**
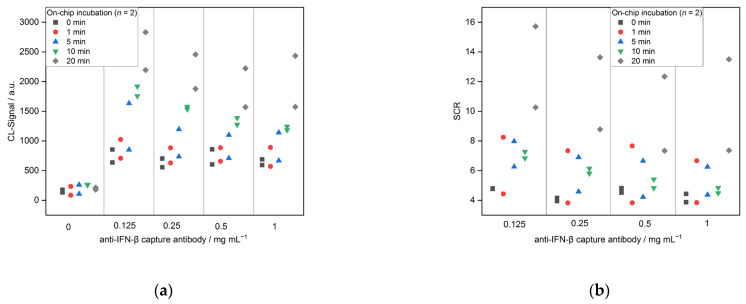
Optimization of incubation in microarray chip for CL-SMIA (*n* = 2): (**a**) CL-signals and (**b**) SCRs.

The next step in the CL-SMIA was the incubation of the pre-incubated sample mixture on the microarray chip, so the IFN-β-DAB complex can interact with the immobilized CABs. Five different incubation times (0, 1, 5, 10 and 20 min) were tested. Figure 6 shows a sharp increase in CL signals with increasing incubation times from 856 and 638 for 0 min to 2833 and 2195 for 20 min (both for 0.125 mg mL^−1^ CAB). The negative control signals on the other hand all stayed at the same level, between 86 and 264. These results show that the SCRs showed the same trend increasing from 4.8 to 15.7 and 10.3, respectively. The longer the incubation time, the more time the complex has to interact with the immobilized CABs. As a result, 20 min were chosen for further measurements.

**Figure 7:**
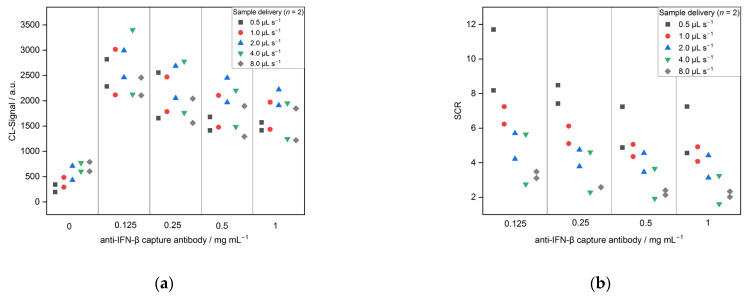
Optimization of sample delivery over the chip for CL-SMIA (*n* = 2): (**a**) CL-signals and (**b**) SCRs.

Figure 7 shows the optimization of the flow rate used to deliver the sample over the flow cell of the chip inside the MCR-R. Five different flow rates were tested (0.5, 1.0, 2.0, 4.0 and 8.0 μL s^−1^). The results show that the signal for the control increased from 345 and 195 for 0.5 μL s^−1^ to 791 and 606 for 8.0 μL s^−1^. While for the higher flow rates, the antigen-antibody complex has less time to bind on the immobilized CAB. Likewise, the BSA present in the sample has less time to bind unspecifically on the microarray surface and, therefore, might have led to an increase in the CL signal for the negative control. Overall, the signal-to-control ratio decreased for higher flow rates from 8.2 and 11.7 for 0.5 μL s^−1^ to 3 and 3.5 for 8.0 μL s^−1^ (both for 0.125 mg mL^−1^ CAB). Hence, the flow rate of 0.5 μL s^−1^ was kept for all further experiments.

**Figure 8:**
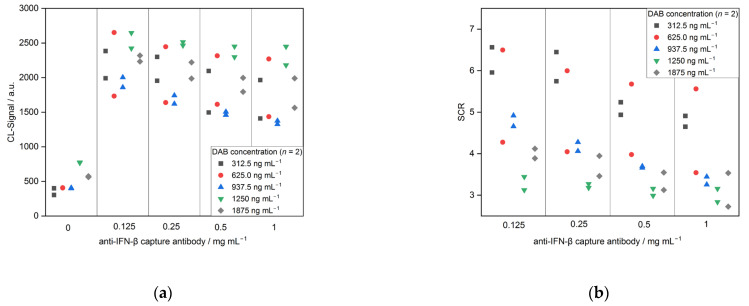
Optimization of DAB concentration for CL-SMIA (*n* = 2): (**a**) CL-signals and (**b**) signal-to-control ratio.

As a last optimization step, the concentration of DAB in the sample-DAB-mix was examined with five concentrations (312.5, 625.0, 937.5, 1250 and 1875 ng mL^−1^). The results are depicted in Figure 8. For the CL signals, no trend can be observed. Only for the negative controls, the CL signals for 1250 and 1875 ng mL^−1^ (approx. 770 and 570) were higher than for the other three concentrations (all approx. 400). Looking at the SCRs, the highest ratios were obtained for 312.5 ng mL^−1^ with 6.6 and 6.0 (0.125 mg mL^−1^ CAB). An explanation for better results with a lower DAB concentration is the steric hindrance for the interaction with the immobilized CAB through an excess of the DABs binding to IFN-β.

#### 3.2.2. Calibration

**Figure 9:**
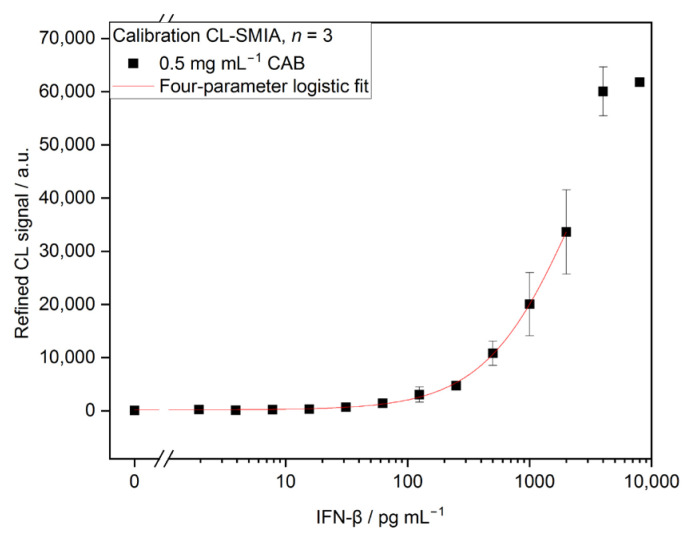
Calibration curve of CL-SMIA for IFN-β with an immobilized CAB concentration of 0.125 mg mL^−1^ (*n* = 3, LOD 4.53 pg mL^−1^, EC50 3860 pg mL^−1^). IFN-β concentrations of 2000 and 4000 pg mL^−1^ were excluded from fit due to CCD camera saturation.

With this optimized assay protocol (312.5 ng mL^−1^ DAB, 45 min pre-incubation, 20 min on-chip incubation, sample delivery over chip with 0.5 μL s^−1^, 1:2500 dilution of strep-HRP), calibration for IFN-β was performed. Refined CL signals (according to Equation (4)) were plotted in Figure 9 semi-logarithmically (*y*-axis linear, *x*-axis logarithmic) versus the concentration of human IFN-β standard and fitted with a four-parameter logistic fit (Equation (1)). As signals for 2000 and 4000 pg mL^−1^ were in the saturation of the CCD camera, these values were excluded for fitting. The best calibration was obtained with 0.125 mg mL^−1^ CAB, resulting in a LOD of 4.53 pg mL^−1^ and an EC50 of 3860 pg mL^−1^. Other fittings with corresponding LODs and EC50s are shown in Supplementary Materials Figure S1b.

(4) $$\mathrm{refined}\;\mathrm{CL}\;\mathrm{signal}=\mathrm{CL}\;\mathrm{signal}\;\left(\mathrm{CAB}\right)-\mathrm{CL}\;\mathrm{signal}\;\left(\mathrm{negative}\;\mathrm{control}\right)$$

### 3.3. Measurements in Nasal Samples

To test the assays in a real matrix, nasal samples were spiked with different concentrations (5.98, 12.04, 23.87 and 58.49 pg mL^−1^) of IFN-β and were measured in triplicates with both ELISA and CL-SMIA. For the 5.98 and 12.04 pg mL^−1^, no recoveries could be calculated for CL-SMIA, while for ELISA, recoveries of 71 ± 6 and 87 ± 2% were found. For 23.87 and 58.49 pg mL^−1^, ELISA showed recoveries of 57 ± 5% and 54 ± 2%, respectively. The CL-SMIA had lower recoveries with 11 ± 13% (*n* = 2) and 14 ± 6%, respectively. The lower recoveries for CL-SMIA compared to ELISA can be reasoned by interactions between DAB and the matrix during pre-incubation, which is excluded for ELISA. Overall, the low recoveries for both assays show the matrix’s influences.

### 3.4. Comparison of ELISA and CL-SMIA

For both ELISA and CL-SMIA, the same antibodies from a commercially available kit (R&D Systems Human IFN-β DuoSet ELISA) were used. The LODs for ELISA and CL-SMIA are in the same range with 1.60 pg mL^−1^, and 4.53 pg mL^−1^, respectively. Both assays work in a sandwich format, meaning IFN-β is captured by the immobilized anti-human IFN-β CAB and detected by the biotinylated anti-human IFN-β DAB. The main difference in the assay formats is the IFN-β-DAB complex, which is formed during the pre-incubation step of CL-SMIA. This complex then binds to the CAB immobilized on the microarray chip, whereas the ELISA IFN-β first interacts with CAB immobilized inside the microtiter well, before—and after blocking—the DAB binds. The difference in recoveries from the real samples might come from this difference in sample incubation.

We were able to produce foil-based microarray chips with a total cost of 1.60 EUR per microarray chip (Table 3). The biggest part, thereof, is represented by the assembly of the chip, more precisely the POM carrier plate and the double-sided adhesive. Since currently the POM carriers are bought from a small handcraft business, where they are cut into shape and outlets are drilled, the price of one carrier at the moment lies at 1.15 EUR. Further improvement of the manufacturing process by, for example, injection moulding will decrease the price of the carriers and, therefore, of the microarray chips. This is combined with the costs for measuring, adding up to 2.37 EUR for one CL-SMA measurement. For ELISA, measuring one 96-well microtiter plate costs 86.19 EUR and, therefore, one well is equal to 0.90 EUR. This means that, for a high number of samples and only for one biomarker, ELISA is more affordable than the CL-SMIA. In this proof-of-concept study since only one antibody was immobilized on the microarray chip. However, up to 20 different antibodies could be immobilized, enabling the simultaneous detection of multiple biomarkers. Since, for ELISA, a separate measurement is required for each biomarker, CL-SMIA will be more affordable for the simultaneous detection of at least three different biomarkers.

One aspect affecting the assay costs is the used amount of antibodies. While the CAB concentration for CL-SMIA is 63 times higher than for ELISA, its small spot volume of 0.9 nL, compared to 100 μL used in ELISA, requires a 362 times higher CAB amount for ELISA, compared to a spot row for the microarray chip (five spots). For DAB, on the other hand, the CL-SMIA requires a five times higher concentration and a three times higher amount per measurement than the ELISA. However, considering the total antibody amounts, one ELISA well costs only 0.65 EUR for the antibodies, while one microarray chip (one immobilized CAB row) costs only 0.07 EUR.

Additionally, the CL-SMIA only requires half of the sample volume as ELISA. The similar LODs of both assays prove that there is no disadvantage in using less sample volume. The smaller sample volume allows for the possibility of more measurements per sample. It also might allow for smaller volumes used for elution for nasal sample collection, which possible could lead to higher concentrations of eluted biomarkers. However, with the aim of further multiplexing the CL-SMIA, the proportion is shifted even more in favour of the CL-SMIA.

In terms of throughput, ELISA has a big advantage over CL-SMIA, as one microtiter plate contains 96 wells and several plates can be performed in one day. This allows for hundreds of sample measurements in one day, even if plate-wise calibration is performed. For the CL-SMIA, on the other hand, only 24 measurements are possible in one working day with an assay time of 1 h 15 min and staggering in time. This sums up to 25 h for 96 measurements if measured continuously or for four working days, while ELISA only needs 6 h (with pipetting steps), if the coating overnight is neglected (Table 4). Furthermore, one measurement needs this 6 h for ELISA, which makes the CL-SMIA more suitable for point-of-care applications. For simplifications, the plate-wise calibration for ELISA was neglected in these considerations.

## 4. Conclusions

In this proof-of-concept study, a flow-based CL-SMIA for the quantification of IFN-β in nasal secretions was developed using a commercially available ELISA antibody kit. For comparison, sandwich ELISA was performed with the same antibody set. The LODs for both assays are in the same size range, indicating similar assay efficiency. The short assay time of the CL-SMIA, below 2 h, and the possibility of staggering the time allows for the fast measuring of small sample sizes. Currently, the costs for CL-SMIA exceed the costs for ELSA, but with the further improvement of producing the microarray chips and the further multiplexing of the assay, a low cost can be achieved. In summation, we were able to show the concept of a flow-based CL-SMIA for the detection of biomarkers, which holds great potential for multiplexed screening of biomarkers in nasal secretions as a point-of-care application.

## Figures and Tables

**Figure 4:**
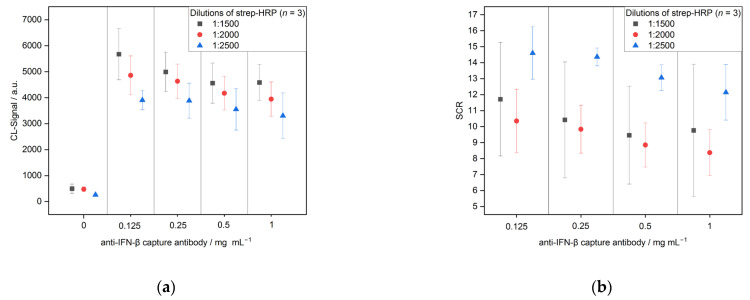
Optimization of the strep-HRP dilutions for CL-SMIA (*n* = 3): (**a**) chemiluminescence (CL)-signals and (**b**) signal-to-control ratios (SCRs).

**Table 1:** Parameters for the chemiluminescence sandwich microarray immunoassay (CL-SMIA) for optimization experiments and final protocol.

Optimization of	Pre-Incubation/min	Incubation Chip/min	DAB /ng mL^−1^	Sample Delivery /μL s^−1^	Dilution Strep-HRP
Strep-HRP	30	5	625.0	0.5	1:1500, 1:2000, 1:2500
Pre-incubation	15, 30, 45, 60	5	625.0	0.5	1:2500
Incubation chip	45	0, 1, 5, 10, 20	625.0	0.5	1:2500
Sample delivery	45	20	625.0	0.5, 1.0, 2.0, 4.0, 8.0	1:2500
DAB concentration	45	20	312.5, 625.0, 937.5, 1250, 1875	0.5	1:2500
Final	45	20	312.5	0.5	1:2500

**Table 2:** Measuring program for the flow-based CL-SMIA on the MCR-R (Microarray Chip Reader-Research).

Process	Volume/μL	Time/min	Comments
Pre-incubation	Sample 50	45	
	DAB 1.25		
	BSA 6.9		
Injection into chip	60	20	With pipette
Insert chip into device			
	**Volume/μL**	**Flow rate/μL s^−1^**	
Direct sample over chip	50	0.5	
Wash chip	1500	325	2 increments, pause 1 s
Incubate strep-HRP	160	50	
	600	2	
Wash chip	1500	325	2 increments, pause 1 s
Add CL reagents	400	100	Mixture 1:1
Take image			60 s exposure
Flush device	2500	500	Sample loop
	2500	500	Sample way
	2500	500	Chip (extra washing chip)

**Table 3:** Costs for the production of one microarray chip, split into different steps of production.

	Cost	Comments
PC-chip surface	0.21 €	PC foil and coating
Spotting	0.002 €	Spotting buffer and CAB antibody
Assembly of microarray chip	1.38 €	POM carrier and adhesive
Total	1.60 €	

**Table 4:** Comparison of CL-SMIA and ELISA regarding assay time. For sample and DAB incubation the assay steps for CL-SMIA change compared to ELISA since DAB and sample are first pre-incubated (1) and then incubated on the microarray chip (2). Time is for one ELISA 96-well microtiter plate or for one microarray chip for CL-SMIA. A total of 96 measurements correspond to one plate of ELISA and 96 continuous, stacked CL-SMIA measurements, respectively.

Assay Step		ELISA	CL-SMIA
Sample incubation		2 h	(2) 20 min on-chip
Blocking		1 h	-
DAB incubation		2 h	(1) 45 min pre-incubation
Detection		40 min	10 min
Total	1 measurement	5 h 40 min	1 h 15 min
	Duplicate	5 h 40 min	1 h 30 min
	96 measurements	5 h 40 min	25 h

## Data Availability

Data will be made available upon reasonable request.

## Supplementary Materials

The following supporting information can be downloaded at: https://www.mdpi.com/article/10.3390/bios13070670/s1, Table S1. ELISA optimization results. Figure S1. Results for calibration experiments of CL-SMIA for IFN-β.
