# Supplementary material for: Flow-Based CL-SMIA for the Quantification of Protein Biomarkers from Nasal Secretions in Comparison with Sandwich ELISA
Source: Biosensors (Basel). 2023 Jun 22;13(7):670. doi: 10.3390/bios13070670 (PMC10377473; doi:10.3390/bios13070670)
Supplement: Supplementary file 1 [file biosensors-13-00670-s001.zip › biosensors-2409416-supplementary.pdf]

Supplementary information

Table S1. ELISA optimization results.

Shown are the sample-to-blank ratios of absorbance at 450 nm. A high sample-to-blank ratio, especially at low IFN-β concentration is a good indication of later assay performance. Two different blocking agents (casein and bovine serum albumin (BSA)), four different detection antibody (DAB) concentrations and three different capture antibody (CAB) concentrations were tested (n = 2). The combination with the highest ratios was chosen and highlighted in this table.

**Blocking agent**	**DAB / ng mL^{-1}**	**CAB / µg mL^{-1}**	**IFN-β / pg mL^{-1}**					
			500		62.5		1.95	
1% Casein	500	2.0	35.8	35.2	6.5	6.7	0.8	1.1
		4.0	42.6	40.6	7.3	7.5	1.3	1.4
		6.0	37.5	36.4	6.7	6.8	1.1	1.6
	250	2.0	57.1	58.3	10.9	11.0	1.2	1.5
		4.0	44.1	42.2	7.7	7.9	1.1	1.2
		6.0	28.2	28.7	5.2	5.3	0.8	0.8
	125	2.0	59.5	83.6	15.2	15.2	1.4	1.3
		4.0	53.2	54.9	10.3	10.5	1.0	1.1
		6.0	30.8	38.7	5.2	6.7	0.9	0.9
	62.5	2.0	73.3	86.5	17.7	17.8	1.8	1.5
		4.0	84.5	82.5	13.4	13.9	1.4	1.5
		6.0	72.2	72.6	12.3	12.4	1.3	1.3
1% BSA	500	2.0	7.0	6.9	1.8	1.7	0.9	0.9
		4.0	6.3	6.1	1.7	1.6	0.9	0.8
		6.0	5.3	5.1	1.5	1.4	0.8	0.8
	250	2.0	9.4	9.2	2.5	2.4	1.0	1.0
		4.0	7.3	7.2	2.1	2.0	0.9	0.8
		6.0	5.6	5.4	1.7	1.7	0.9	0.8
	125	2.0	6.3	8.5	2.2	2.1	0.9	0.9
		4.0	7.3	6.8	2.0	1.9	0.8	0.9
		6.0	4.8	5.2	1.7	1.7	0.8	0.8
	62.5	2.0	7.7	9.3	2.5	2.4	0.9	1.1
		4.0	8.4	8.3	2.1	2.1	0.8	0.8
		6.0	6.4	6.9	2.0	1.8	1.0	1.0

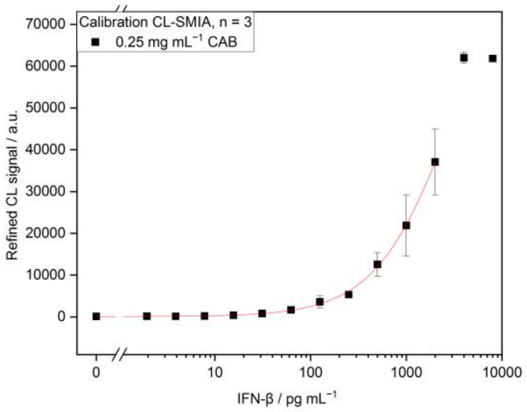

(a)

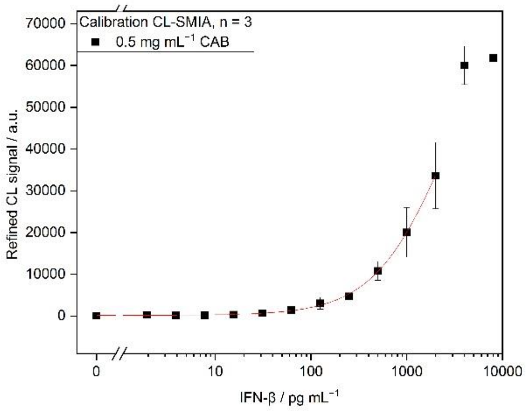

(b)

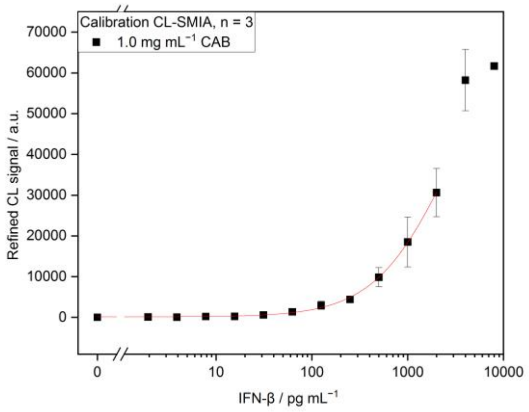

(c)

Figure S1. Results for calibration experiments of CL-SMIA for IFN-β (n = 3).

(a) 0.25 mg mL^{-1}, LOD 20.01 pg mL^{-1}, EC50 3280 pg mL^{-1}; (b) 0.5 mg mL^{-1}, LOD 24.02 pg mL^{-1}, EC50 2567 pg mL^{-1}; (c) 1.0 mg mL^{-1}, LOD 36.82 pg mL^{-1}, EC50 2555 pg mL^{-1}. IFN-β concentrations of 2000 and 4000 pg mL^{-1} were excluded from fit due to CCD camera saturation.
